# Children with mild hyponatremia at the emergency department are at higher risk of hospitalization

**DOI:** 10.1186/s12887-023-04109-8

**Published:** 2023-06-23

**Authors:** Stefano Pintaldi, Alessandro Zago, Carlo Pizzolon, Elena Magni, Giorgio Cozzi, Stefanny Andrade, Egidio Barbi, Alessandro Amaddeo

**Affiliations:** 1 Department of Medicine, Surgery and Health Sciences, University of Trieste, 34137 Trieste, Italy; 2 Institute for Maternal and Child Health IRCCS “Burlo Garofolo”, 34137 Trieste, Italy

## Abstract

**Background:**

Mild hyponatremia is frequently encountered in the pediatric emergency department (PED). Although usually of little clinical concern, its prognostic meaning as a possible marker of more severe disease has not yet been well established.

**Methods:**

We retrospectively analyzed data from children and adolescents who performed a blood sample with plasmatic sodium measurement on admission to the PED of IRCCS “Burlo Garofolo” Pediatric Hospital in Trieste, Italy, in 2019. We compared the rate, length of admissions and laboratory characteristics of patients with hyponatremia to those with normal sodium.

**Results:**

Among 807 subjects, hyponatremia (sodium < 135 mEq/L) was present in 17.6%, being mild (between 130 and 134 mEq/L) in 16.5%. Hyponatremic patients were younger, more frequently males, with an infection diagnosis, mainly of the respiratory tract and viral aetiology. They presented higher C-reactive protein (CRP) levels and erythrocyte sedimentation rates (ESR). Compared to normonatremic individuals, hyponatremic patients presented a higher risk of underlying infection (aOR 2.02; 95%CI 1.33–3.08), hospital admission (aOR 1.72; 95%CI 1.06–2.48), and a hospital stay of > 5 days (aOR 1.99; 95%CI 1.03–3.85). When considering only subjects with mild hyponatremia, we found similar results.

**Conclusion:**

Hyponatremia and mild hyponatremia in the PED are associated with an increased admission rate and extended hospital stays. Mild hyponatremia should be considered a warning sign for a possibly more serious condition.

**Supplementary Information:**

The online version contains supplementary material available at 10.1186/s12887-023-04109-8.

## Introduction

Hyponatremia, defined as a plasma (or serum) sodium level below 135 mEq/L, is commonly encountered in pediatric patients in the emergency department, with a reported incidence of 17–45% [1, 2]. It is the most common electrolyte disorder and can be classified as mild when serum sodium concentration is between 130 and 134 mEq/L, moderate between 129 and 125 mEq/L and severe when below 125 mEq/L [3]. Plasma sodium concentration is fundamental for water balance. Sodium level affects the cellular volume and determines tonicity, thus regulating fluid distribution among different body compartments. The most accounted pathophysiological mechanism suggests the role of fever and proinflammatory cytokines, such as IL-6 and IL-8, in stimulating ADH secretion, eventually leading to hyponatremia [4]. This hypothesis is consistent with studies that show a direct link between plasma sodium concentration and plasma CRP elevation [5]. Hyponatremia causes water to move from the extracellular space to the intracellular compartment, causing cerebral oedema and intracranial hypertension. The latter could arise earlier in children than adults due to the higher brain-to-skull size proportion.

Clinical features of severe hyponatremia include headache, vomiting, loss of consciousness, seizures, and coma. Seizures are typically poorly responsive to anti-epileptic treatment and usually occur when sodium falls below 120 mEq/L. Several causes of hyponatremia exist and can be classified based on volume status.

Several studies on the adult population, including thousands of patients with heart, liver, and pulmonary diseases, reveal that hyponatremia is strongly associated with a more severe disease course and higher overall mortality [4].

To date, studies in the pediatric population are primarily retrospective and focused on specific diseases. According to the literature, hyponatremia is associated with respiratory infections, such as pneumonia and bronchiolitis, urinary tract infections, gastrointestinal infections, meningitis [10], Kawasaki disease, sepsis, and malaria. The association between hyponatremia, respiratory infections, and pneumonia is well established. Hyponatremia is reported to occur in 13.5–45.4% of cases, mainly mild and related to higher fever, elevated inflammatory markers, higher leucocyte count and hospitalization rates [2, 6–11]. The same is true for children with bronchiolitis, in whom hyponatremia can occur in up to 57%, mostly in children aged under six months. Among those infants who enter the ICU, sodium below 135 mEq/L is a risk factor for a more extended stay and mechanical ventilation.

Data on the association between hyponatremia and age are controversial. Mazzoni studied 400 children with community-acquired infections and found that those with hyponatremia were younger than controls [12]. Conversely, Sung observed the opposite in a larger sample of children with respiratory infections [13].

Lehtiranta et al. published a recent registry-based cohort study involving 46,518 children who accessed PED demonstrating that hyponatremia was an independent risk factor for hospitalization and the need for PICU treatment. In this series, only moderate to severe hyponatremia carried a higher risk of neurological symptoms and deaths [14].

Previous studies demonstrated that hyponatremia not only accompanies but also correlates with the severity of systemic inflammatory response in pneumonia, acute appendicitis, and Kawasaki disease [15]. Remarkably, a meta-analysis that included 81 studies in pediatric and adult patients demonstrated an association of hyponatremia with increased mortality, with an overall RR of 2.60 [16].

Published data on hyponatremia on admission in a pediatric emergency department (PED) are scarce and mainly based on already hospitalized patients, with the possible bias of including iatrogenic hyponatremia due to overuse of hypotonic intravenous fluids [17]. To date, no studies in the pediatric emergency setting analyze the role of hyponatremia as an independent risk factor for a more severe disease course or a more extended hospitalization, independent of the aetiology. Furthermore, while the role of severe hyponatremia is well established, less is known about the relevance of mild hyponatremia. In fact, to the best of our knowledge, only one study specifically addressed the implications of mild hyponatremia only in the ED setting. [13]

This study investigates whether mild hyponatremia in children referred to a PED is a risk factor for a more severe disease course regarding admission risk, length of hospitalization and elevation of inflammatory markers.

## Methods

This is a retrospective with a case control comparison on a part of the data presented to the PED of IRCCS “Burlo Garofolo” Pediatric Hospital in Trieste, Italy, from January 1st 2019, to December 31st 2019.

The PED is the only facility in the setting of a third-level pediatric research and teaching hospital in an area of 260,000 inhabitants. The average number of accesses per year is 24,000, with an admission rate of 3.5% in short observation and 2.5% in the ward.

We recruited all the subjects between the ages of three months and seventeen years who had a blood sample, including serum sodium concentrations, taken on admission, according to the evaluation and decision of the treating physician.

Hyponatremia was classified as mild when serum sodium concentration was between 130 and 134 mEq/L, moderate between 129 and 125 mEq/L and severe when below 125 mEq/L.

As far as the length of stay parameter was considered, the cut-off was established according to the average length of stay of the normonatremic patients.

For each subject, we gathered the following data: gender, age at admission, degree of hyponatremia (mild, moderate, severe), inflammatory markers plasmatic concentrations (C-reactive protein CRP and erythrocytes sedimentation rate ESR), length of hospitalization and discharge diagnosis. We classified the diagnosis into six macro-groups: infections, inflammatory diseases, oncohematological diseases, musculoskeletal and soft tissue diseases, central nervous system (CNS) diseases, and others. We further subdivided the infections macro-group into the upper respiratory tract, lower respiratory tract, gastrointestinal, genito-urinary, musculoskeletal and soft tissues, and others. When available, the aetiology of infections was recorded (viral or bacterial).

Ethical Committee approval was not requested according to the Italian Law since General Authorization to Process Personal Data for Scientific Research Purposes (Authorization no. 9/2014) declared that retrospective archive studies that use ID codes, preventing the data from being traced back directly to the data subject, do not need ethics approval [18].

According to the Research Institute policy, all parents at admission signed an informed consent to authorize the anonymous use of data.

### Statistical analysis

Data were reported as numbers, percentages for categorical variables, and mean and standard deviation (or median and interquartile range) for continuous variables. We compared hyponatremic (cases) and normonatremic (controls) subjects using the chi-square test - or Fisher’s exact test when appropriate - for categorical variables. In contrast, the Student’s t-test or Mann-Whitney test were employed respectively for normally and not-normally distributed quantitative variables. The differences were considered statistically significant for p-values below 0.05.

Logistic regression analysis assessed risk factors for hyponatremia and mild hyponatremia and evaluated the chance of admission, infection and length of stay (> 5 days) for hyponatremic and mild hyponatremic patients. The controls were matched for age with a 2:1 control-to-case ratio, and analyses were adjusted for sex to assess the risk of admission, infection and extended hospitalization. All statistical analysis was performed using R Software, Version 4.1.1 (R Foundation for Statistical Computing, Vienna) and STATA Statistical Software, Version 17 (2021. College Station TX, StataCorp LLC).

## Results

We identified a total of 824 children and adolescents for data collection. Of those, 17 patients were excluded due to lack of documentation/ incomplete information/ laboratory errors in sample analysis, resulting in 807 patients included for analysis. All patients had blood sampling with sodium available before starting any infusion or supplementation of oral rehydration solutions. Of these, one hundred and forty-two children (17.6%) were hyponatremic on admission; 133 presented mild hyponatremia (16.5%), while patients with moderate and severe hyponatremia were 7 (0.9%) and 2 (0.2%) respectively.

Table 1 reports the differences between hyponatremic and normonatremic subjects.

**Table 1:** Characteristics of patients with hyponatremia (sodium <134 mEq/L) compared to those with normal sodium

Whole sample	Normonatremia (N = 665)	Hyponatremia (N = 142)	p-value
**Age** years, m ± SD	9.6 ± 5.9	5.3 ± 4.5	**< 0.001**
**Sex** n° (%)			**0.001**
Females	342 (51.4)	52 (36.6)	
**Diagnosis** n° (%)			
**Infection**	271 (40.8)	93 (65.5)	**< 0.001**
** Organ system**			
Upper respiratory	29 (4.4)	13 (9.2)	**0.020**
Lower respiratory	61 (9.2)	26 (18.3)	**0.001**
Gastrointestinal	54 (8.1)	7 (4.9)	0.192
Genito-urinary	10 (1.5)	5 (3.5)	0.106
Osteomuscular/soft tissue	15 (2.3)	4 (2.8)	0.759
** Etiology**			
Viral	68 (10.2)	27 (19.0)	**0.003**
Others	34 (5.1)	11 (7.8)	0.214
**Inflammatory**	18 (2.7)	6 (4.2)	0.334
**Onco-haematological**	11 (1.6)	3 (2.1)	0.722
**Osteomuscular**	58 (8.7)	3 (2.1)	**0.005**
**CNS**	67 (10.1)	15 (10.6)	0.861
**Others**	240 (36.1)	22 (15.5)	**< 0.001**
**Outcome** n° (%)			**0.018**
Discharge	447 (67.5)	79 (55.6)	
Admission	208 (31.4)	62 (43.7)	
Transfer	7 (1.1)	1 (0.7)	
**Length of hospitalisation** days,med (IQR)	4 (3–6)	5 (3–8)	**0.018**
**ESR** mm/h, med (IQR)	17 (5–37)	48 (22–78)	**< 0.001**
**CRP** mg/L, med (IQR)	4.3 (0.7–18.8)	31 (0.4–90.5)	**< 0.001**

In the hyponatremic group, the subjects were, on average, 4.3 years younger than the normonatremic ones, with a mean age of 5.3 years, and most of them were males (63.4%). Ninety-three (65.5%) patients with hyponatremia had a diagnosis of infection, a percentage much higher than the control group (40.8%). The most common sites were the upper (9.2%) and lower (18.3%) respiratory tract, and viral aetiology was the most common cause (19%). Almost half of the patients with low blood sodium (43.7%) were admitted to the hospital, 12.3% more than the control group, and they were discharged almost one and a half days later than the control. The mean CRP level was more than three times higher in the hyponatremic patients compared to normonatremic patients (61.3 mg/L vs. 19.9 mg/L). Similarly, the ESR doubled in normonatremic individuals (53.2 mm/h vs. 26.2 mm/h).

Considering only subjects with sodium 134 − 130 mEq/L, we found similar results (Supplementary Table).

Table 2 reports multiple logistic regression analyses for hyponatremia and mild hyponatremia-associated risk factors. A younger age, male sex, and higher levels of ESR and CRP were all independent risk factors for hyponatremia.

**Table 2:** Risk factors for hyponatremia and mild hyponatremia analyzed with logistic regression

	Hyponatremia (n = 142)			Mild hyponatremia (n = 133)			
*Risk Factor*	**aOR (95% CI)**	**p-value**		**aOR (95% CI)**	**p-value**		
**Age (younger)**	1.15 (1.08–1.20)	**< 0.001**		1.13 (1.07–1.20)	**< 0.001**		
**Sex (male)**	1.85 (1.07–3.19)	**0.028**		2.00 (1.15–3.45)	**0.013**		
**ESR (higher)**	1.01 (1.00–1.02)	**0.012**		1.01 (1.00–1.02)	**0.005**		
**CRP (higher)**	1.01 (1.00–1.01)	**0.001**		1.00 (1.00–1.01)	0.127		

**Fig. 1:**
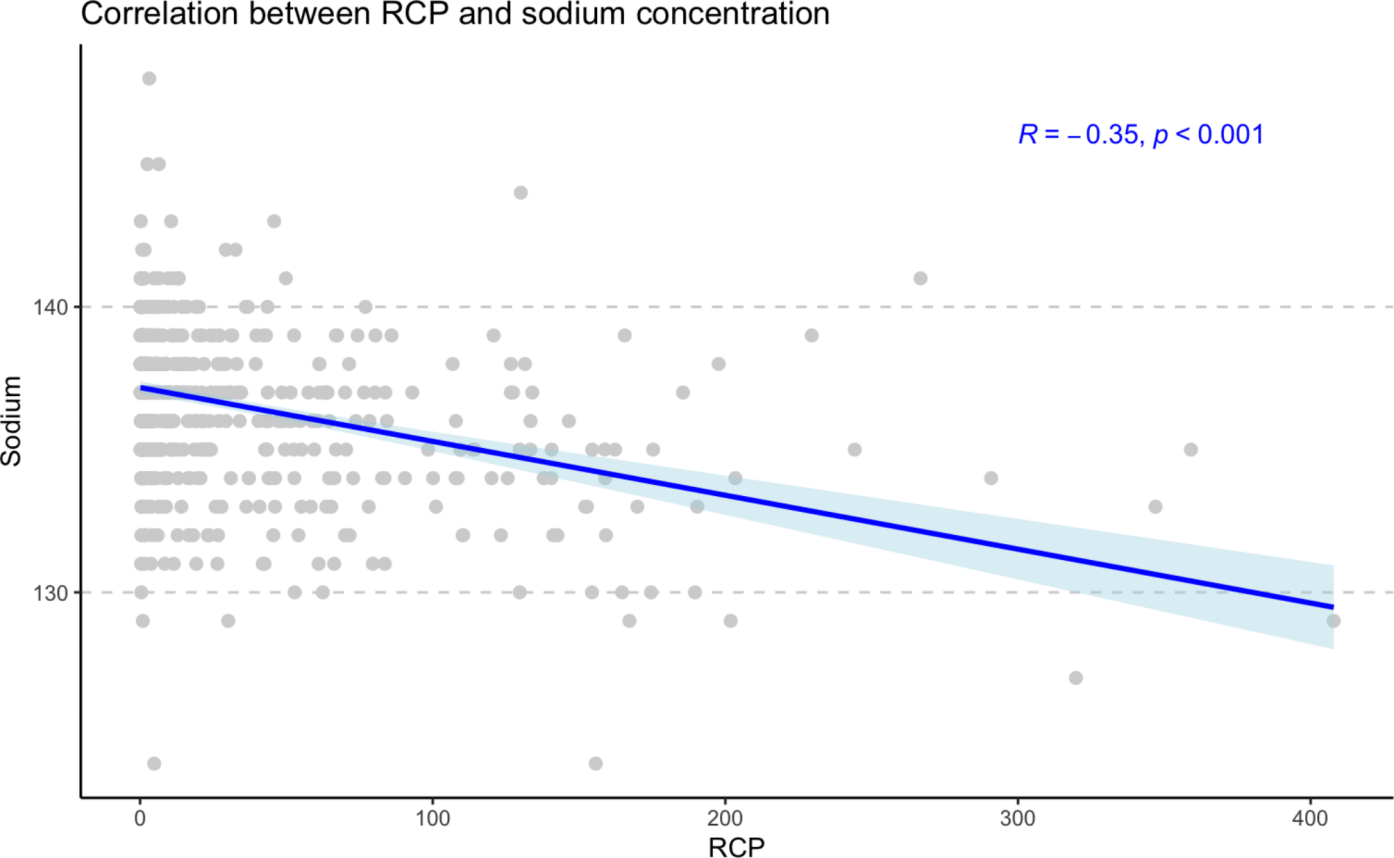
Negative linear correlation between CRP and plasmatic sodium concentration, Pearson’s *r* of -0.351 (p < 0.001)

Figure 1 shows Pearson’s negative linear correlation between higher levels of CRP and hyponatremia (r -0.351; p < 0.001).

Table 3 reports the logistic regression analysis performed by matching individuals for age and adjusting the odds ratio for sex. When compared to normal blood sodium values, both hyponatremia and mild hyponatremia were associated with an increased risk of hospital admission (aOR 1.72; 95%CI 1.13–2.62 and aOR 1.62; 95%CI 1.06–2.48 respectively) and a diagnosis of infection (aOR 2.02; 95%CI 1.33–3.08 and aOR 2.05; 95%CI 1.34–3.15 respectively), while only hyponatremia was associated with hospital stays longer than five days (aOR 1.99; 95%CI 1.03–385 and aOR 1.79; 95%CI 0.92–3.48 respectively). Finally, we performed a comparison between subjects with mild hyponatremia and subjects with moderate to severe hyponatremia that showed a higher value of CRP in the latter group (161.0 ± 148.8 mg/L vs. 54.7 ± 64.7 mg/L; p < 0.001 not shown in tables).

**Table 3:** Risk of hospital admission, more extended hospital stays and underlying infection for subjects with hyponatremia (n°142) and mild hyponatremia (n°133) compared to normonatremic individuals. Controls were matched for age with a 2:1 control-to-case ratio, and analyses were adjusted for sex

*Outcome*		aOR (95% CI)		p-value	
Hyponatremia vs. normonatremia					
**Risk of admission**	1.49 (1.01–2.20)		**0.043**		
**Risk of infection**	1.95 (1.31–2.90)		**0.001**		
**Stay ≥ 5 days**	1.84 (1.01–3.34)		**0.045**		
Mild hyponatremia vs. normonatremia					
**Risk of admission**	1.42 (0.95–2.11)		**0.085**		
*Risk of infection	2.00 (1.32–3.00)		**0.001**		
**Stay ≥ 5 days**	1.79 (0.97–3.30)		0.063		

* Risk of infection refers to the risk of infectious versus noninfectious disease

Table 4 reports the characteristics of the nine patients with moderate or severe hyponatremia

**Table 4:** Characteristics of patients with moderate and severe hyponatremia

N°patient	Sex	Age (years)	Plasmatic Na (mEq/L)	Diagnosis	Admission	Length of hospitalization (days)	ESR (mm/h)	CRP (mg/L)
1	M	2	129	Bronchopneumonia	no	/	35	30.1
2	M	2	129	Viral infection, aspecific	no	/	/	0.9
3	F	3	129	Peritonitis	yes	4	106	407.9
4	M	3	127	Pneumonia	yes	3	102	319.8
5	F	3	129	Pneumonia	no	/	59	201.8
6	F	4	124	Encephalitis, myelitis, encephalomyelitis	yes	12	48	155.7
7	M	4	124	Diabetes with ketoacidosis	yes	10	/	4.8
8	F	12	129	Empyema (possible Kawasaki’s disease)	yes	10	120	167.2
9	F	13	128	Diabetes with ketoacidosis	no	/	/	/

## Discussion

This study confirms that hyponatremia is a common condition in the PED, occurring in 17.6% of children who undergo a blood sampling, representing a risk factor for admission and a more extended hospital stay.

These data show that hyponatremia in a PED is primarily mild and presents mainly in younger children with respiratory infections of viral origin. Males were more likely to have hyponatremia, although this may be explained by the higher incidence of viral infections in males in the first year of life [19].

The main finding of this study is that we reached the same conclusions in the mild hyponatremia subgroup analysis. While moderate to severe hyponatremia carries a well-known risk of severe disease, mild hyponatremia is a common finding that emergency physicians may quickly seek. Indeed, this series shows that a plasmatic sodium concentration of 134 − 130 mEq/L is associated with a more elevated risk of severe infections and a higher hospitalization rate. It suggests that clinicians should carefully consider this laboratory sign. In their milestone study, Lethiranta and colleagues [13] revealed that mild hyponatremia is related to worse outcomes, with even higher OR than our series. However, in that paper, 15% of children already received an iv infusion of moderately hypotonic fluid therapy with 60–80 mmol/L of sodium. Our series is the first to refer to mild hyponatremia in a selected cohort of children not receiving any previous infusion.

From a pragmatic perspective, the study design does not allow for the inference that an additional cause of hyponatremia would worsen patient outcomes. However, the awareness of the potential risks related to mild hyponatremia at admission is relevant, reminding physicians of the eventual iatrogenic dangers related to the use of hypotonic fluids in children with acute conditions [17]. Hyponatremia occurs more frequently in infants with respiratory infections such as bronchiolitis and pneumonia, which are well-known causes of a syndrome of inappropriate ADH secretion. In this setting, intravenous hypotonic fluids and overhydration should be carefully avoided since they can lower the sodium further to harmful levels [20].

The study’s limitations are the retrospective monocentric design and the fact that sodium levels were assessed according to the attending physician’s clinical decision. The number of patients with severe hyponatremia was too limited to allow a comparison between severe and mild cases. Furthermore, no clinical details such as hypoxia, weight loss or gain, and days of disease were analyzed. Finally, we did not investigate mechanisms underlying hyponatremia since it was beyond the scope of our study.

## Conclusion

This study shows that mild hyponatremia in the PED is associated with younger age, respiratory infections, an increased odd ratio of admission and underlying infection. These data suggest that ED physicians should consider mild hyponatremia as a red flag for a more relevant condition. More data are needed to confirm these results.

## Electronic supplementary material

Below is the link to the electronic supplementary material.

Supplementary Material 1

## Data Availability

The datasets used and/or analyzed during the current study are available from the corresponding author upon reasonable request.
