# Supplementary material for: Children with mild hyponatremia at the emergency department are at higher risk of hospitalization
Source: BMC Pediatr. 2023 Jun 23;23:318. doi: 10.1186/s12887-023-04109-8 (PMC10288815; doi:10.1186/s12887-023-04109-8)
Supplement: Supplementary file 1 — Supplementary Material 1 [file 12887_2023_4109_MOESM1_ESM.docx]

|  | **N=807** |
| --- | --- |
| **Age** years, m ± sd | 8.9 ± 5.9 |
| **Sex**, n (%) |  |
| Males | 413 (51.2) |
| Females | 394 (48.8) |
| **Diagnosis**, n (%) |  |
| **Infection** (tot.) | 364 (45.1) |
| **▪ Organ system** |  |
| Upper respiratory | 42 (5.2) |
| Lower respiratory | 87 (10.8) |
| Gastrointestinal | 61 (7.6) |
| Genito-urinary | 15 (1.8) |
| Osteomuscolar/soft tissue | 19 (2.3) |
| **▪ Etiology** |  |
| Viral | 95 (11.8) |
| Others | 45 (5.6) |
| **Inflammatory** | 24 (3.0) |
| **Onco-haematological** | 14 (1.7) |
| **Osteomuscular** | 61 (7.6) |
| **CNS** | 82 (10.1) |
| **Others** | 262 (32.5) |
| **Outcome**, n (%) |  |
| Discharge | 526 (65.4) |
| Admission | 270 (33.6) |
| Transfer | 8 (1.0) |
| **Lenght of hospitalisation** days, med (IQR) | 4 (3 - 6) |
| **ESR** mm/h, med (IQR) | 20 (6 - 49) |
| **CRP** mg/L, med (IQR) | 6 (0.8 - 28.5) |
|  |  |

Table (supplement). Characteristics of patients with mild hyponatremia (sodium 134-130 mEq/L) compared to those with normal sodium.

*refers to inflammation, rheumatic diseases, trauma
